# VINCENT: A randomized-controlled trial evaluating venetoclax plus azacitidine versus intensive chemotherapy in patients with newly diagnosed, *NPM1*-mutated AML

**DOI:** 10.1007/s00277-025-06496-7

**Published:** 2025-07-09

**Authors:** Lydia Kretschmer, Leo Ruhnke, Christoph Schliemann, Lars Fransecky, Björn Steffen, Martin Kaufmann, Andreas Burchert, Christoph Schmid, Maher Hanoun, Tim Sauer, Klaus H. Metzeler, Kerstin Schäfer-Eckart, Mathias Hänel, Martina Crysandt, Paul Jäger, Stefan W. Krause, Christine Dierks, Stefan Klein, Nadia Maguire, Lukas P. Frenzel, Veit L. Bücklein, Wolfgang Blau, Ulrich Kaiser, Kai Wegehenkel, Alexander Höllein, Ruth Seggewiss-Bernhardt, Wenke Markgraf, Frank Fiebig, Anna Harig, Katharina Schmidt-Brücken, Christian Thiede, Jan Moritz Middeke, Richard Dillon, Claudia D. Baldus, Hubert Serve, Karsten Spiekermann, Wolfgang Hiddemann, Richard F. Schlenk, Carsten Müller-Tidow, Martin Bornhäuser, Christoph Röllig

**Affiliations:** 1 Department of Internal Medicine I, University Hospital Dresden, TU Dresden, Fetscherstraße 74, Dresden, 01307 Germany; 2 Department of Medicine A, University Hospital Münster, Münster, Germany; 3 Department of Hematology and Oncology, University Hospital Schleswig- Holstein, Campus Kiel, Kiel, Germany; 4 Department of Internal Medicine II, University Hospital Frankfurt, Frankfurt, Germany; 5 Robert Bosch Hospital, Stuttgart, Germany; 6 Department of Internal Medicine, Hematology, Oncology and Immunology, University Hospital Marburg, Marburg, Germany; 7 Department of Internal Medicine II, Hematology and Oncology, University Hospital Augsburg, Augsburg, Germany; 8 Department of Hematology and Oncology, University Hospital Essen, University of Duisburg-Essen, Essen, Germany; 9 Department of Internal Medicine V, University of Heidelberg, Heidelberg, Germany; 10 Department of Internal Medicine I, Hematology and Cellular Therapy, University Hospital Leipzig, Leipzig, Germany; 11 Department of Internal Medicine V, Klinikum Nürnberg, Paracelsus Medical University, Nuremberg, Germany; 12 Department of Internal Medicine III, Klinikum Chemnitz, Chemnitz, Germany; 13 Department of Hematology, Oncology, Hemostaseology and Stem Cell Transplantation, Medical Faculty, Center for Integrated Oncology Aachen, Bonn Cologne Duesseldorf, Aachen, Germany; 14 University Hospital of Düsseldorf, Düsseldorf, Germany; 15 Department of Internal Medicine V, University Hospital Erlangen, Erlangen, Germany; 16 University Hospital Halle, Halle, Germany; 17 University Medical Centre Mannheim, Mannheim, Germany; 18 Barmherzige Brüder Hospital, Regensburg, Germany; 19 Department I of Internal Medicine, Faculty of Medicine and Cologne University Hospital, Center for Integrated Oncology Aachen, University of Cologne, Bonn, Cologne, Düsseldorf, Cologne, Germany; 20 LMU Hospital, Munich, Germany; 21 Department of Internal Medicine III, Hematology, Oncology and Palliative Care, Helios Dr. Horst Schmidt Hospital Wiesbaden, Wiesbaden, Germany; 22 Department of Hematology, Oncology and Immunology, St. Bernward Hospital, Hildesheim, Germany; 23 University Hospital OWL/Bielefeld Hospital, Bielefeld, Germany; 24 Department of Internal Medicine III, Hematology and Oncology, Rotkreuzklinikum München, Munich, Germany; 25 Department of Internal Medicine V, Sozialstiftung Bamberg, Bamberg, Germany; 26 Comprehensive Cancer Center Erlangen-EMN, Erlangen, Germany; 27 AgenDix GmbH, Dresden, Germany; 28 King’s College, London, UK

**Keywords:** AML, *NPM1*, Fit patients, Venetoclax, Azacitidine, Intensive chemotherapy

## Abstract

For younger, medically fit patients with *NPM1*-mutated, *FLT3*-wildtype acute myeloid leukemia (AML) intensive chemotherapy represents standard of care (SOC), with complete remission (CR) rates observed in up to 85% of patients and 5-year overall survival (OS) rates of 40–50%. However, significant toxicity and need for hospitalization pose challenges on patients’ outcome and quality of life (QoL). Venetoclax (VEN) combined with azacitidine (AZA) has demonstrated encouraging efficacy in older, unfit AML patients, achieving high CR/CRi rates and promising OS with lower toxicity. Prospective, randomized data comparing VEN/AZA to SOC in younger, fit patients are currently missing. VINCENT is a randomized-controlled, multicenter, non-inferiority, phase 2 trial (NCT05904106) evaluating VEN/AZA versus SOC in adults aged 18–70 years with newly diagnosed, *NPM1*-mutated, *FLT3*-wildtype AML. Patients medically fit for intensive chemotherapy (ECOG ≤ 2) with adequate organ function are eligible, while patients with relapsed/refractory AML or prior cytotoxic treatment are excluded. A total of 146 patients will be randomized 1:1 to receive either VEN/AZA or SOC. Hematologic remission is evaluated according to ELN 2022 guidelines. The primary endpoint is the modified event-free survival, defined as either primary induction failure, hematologic relapse, molecular failure or death. Secondary endpoints include safety, tolerability, CR/CRi/CRh/CR_MRD−_ rates, MRD kinetics (using *NPM1* RT-qPCR and MFC), relapse-free survival, OS, early mortality, health-related QoL and cumulative health-care-resource use. Patients will be followed up for at least two years post enrollment. The VINCENT trial will be the first study to provide comprehensive prospective data comparing VEN/AZA to SOC, addressing both efficacy and patient-centered outcomes.

## Introduction

Mutations in *NPM1* are among the most common genetic alterations in adult patients with acute myeloid leukemia affecting around 30% of them [1]. Patients with *NPM1*-mutated AML are considered having a favorable prognosis, since *NPM1* mutated disease is known to be chemo-sensitive [2, 3]. Accordingly, the WHO 2022 classification recognizes *NPM1*-mutated AML as a distinct entity, and the ELN 2022 risk stratification classifies patients with *NPM1*-mutated, *FLT3*-ITD-negative AML as favorable risk [3]. Standard of care (SOC) in fit, *NPM1*-mutated, *FLT3*-wildtype AML patients consists of intensive induction chemotherapy (commonly 7 + 3 regimens consisting of cytarabine plus an anthracycline/anthracenedione) with or without gemtuzumab ozogamicin (GO), followed by intermediate-dose cytarabine consolidation (IDAC). This treatment approach leads to complete remissions (CR) in up to 85% of patients and corresponding 5-year overall survival (OS) rates of 40–50% [4, 5]. Even though early mortality with intensive chemotherapy dropped substantially over the past 20 years, treatment-related toxicity is still substantial including severe myelosuppression with infectious complications, organ toxicity and long-term sequelae such as cardiac or neurological morbidity, infertility and secondary neoplasms/malignancies [5–7]. Moreover, each course of chemotherapy usually requires hospitalization contributing to patient discomfort and significant economic burden due to extensive care measures.

In contrast, the combination of venetoclax (VEN) and hypomethylating agents, e.g. azacitidine (AZA), has shown encouraging results in elderly patients not eligible for intensive chemotherapy. Accordingly, VEN/AZA has been FDA/EMA-approved for older AML patients considered ineligible for induction therapy. Administered in an outpatient setting, this regimen shows lower toxicity and offers notable efficacy, particularly in patients with *NPM1*-mutated disease. Here, up to 93% of patients with *NPM1*-mutated AML considered not fit for intensive chemotherapy achieved CR/CRi, corresponding with a 2-year OS rate of 72% [8–10]. The outpatient administration also minimizes hospitalization costs and possibly improves health-related-quality of life.

Interestingly, Lachowiez et al. showed a significant survival advantage for VEN/AZA over intensive chemotherapy retrospectively evaluating a cohort of *NPM1*-mutated, treatment naïve AML patients. Here, the survival benefit could not only be seen in older AML patients, but also in younger, medically fit patients [9]. Also, VEN/AZA is considered effective in younger, medically fit patients with *NPM1*-mutated AML experiencing molecular failure. Sartor et al. reported that 82% of patients (9/11) with MRD-positive CR post intensive induction chemotherapy achieved MRD-negativity after a median number of only two cycles of VEN/AZA [11]. VEN/AZA is also an effective treatment in the relapsed/refractory setting [12]. Stahl et al. showed a response rate (CR/CRi) of 46% for the subgroup of *NPM1*-mutated patients compared to a general response rate (CR/CRi) of 24% [13].

However, while retrospective analyses and single-arm studies suggest that VEN/AZA may compare favorably/non-inferior with SOC/intensive chemotherapy, so far there is very little prospective data directly comparing these regimens in younger patients considered medically fit. Thus, the aim of this phase 2 trial is to evaluate the efficacy, safety and tolerability of VEN/AZA in comparison to the current SOC in fit patients with newly diagnosed, *NPM1*-mutated, *FLT3*-wildtype AML.

## Patients and methods

### Study design and objectives

VINCENT is a randomized-controlled, open-label, multicenter, phase 2 trial evaluating efficacy and safety of VEN in combination with AZA compared to SOC intensive chemotherapy in adult treatment-naïve patients with *NPM1*-mutated AML. Patients will be randomized in a 1:1 fashion stratified by age to receive either VEN/AZA or SOC.

The primary objective of this trial is to compare efficacy of VEN/AZA with intensive standard chemotherapy in newly diagnosed, *NPM1*-mutated AML patients within a non-inferiority assumption. Secondary objectives include the evaluation of safety and tolerability, rates of morphologic and molecular remission, MRD kinetics (molecular response and persistence), relapse-free survival and OS, early mortality, health-care resource use and health-related quality of life.

**Fig. 1:**
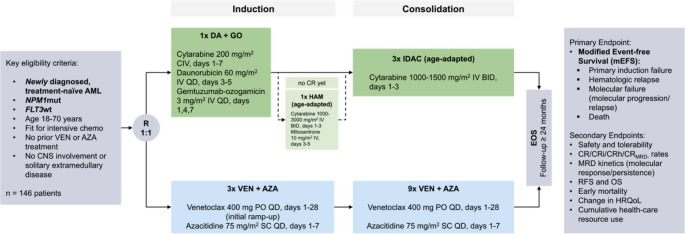
VINCENT trial design. VINCENT is a randomized-controlled, open-label, multicenter, phase 2 trial evaluating efficacy and safety of VEN in combination with AZA compared to SOC intensive chemotherapy in adult treatment-naïve patients with *NPM1*-mutated AML. Patients will be randomized in a 1:1 fashion stratified by age to receive either VEN/AZA or SOC. AML, acute myeloid leukemia; AZA, azacitidine; CNS, central nervous system; CR, complete remission; DA, daunorubicin; EOS, end of study; *FLT3*, FMS-like tyrosine kinase 3; GO, gemtuzumab-ozogamicin; HRQoL, health-related quality of life; IDAC, intermediate-dose cytarabine; *NPM1*, Nucleophosmin 1; RFS, relapse-free survival; OS, overall survival

The study design is outlined in Fig. 1.

In the investigational arm, patients will receive three cycles of VEN (400 mg PO QD, days 1–28, initial ramp-up) and AZA (75 mg/m^2 ^SC QD, days 1–7) for induction, followed by nine additional consolidation cycles of the same regimen for patients in CR/CRi/CRh and absence of molecular failure.

Patients treated within the SOC arm will receive induction therapy with cytarabine, daunorubicin and GO as per the 7 + 3 + GO regimen. Patients not achieving CR after first induction therapy (IT1) will receive age-adapted HAM (high dose cytarabine plus mitoxantrone) for IT2. Patients achieving CR/CRh will receive up to three cycles of age-adapted IDAC for post-remission treatment.

The study (NCT05904106) is conducted in accordance with the Declaration of Helsinki and approved by the institutional review committee of the Technische Universität (TU) Dresden (EK13012023). Currently 25 German Study Alliance Leukemia (SAL) and AML Cooperative Group (AMLCG) centers are actively recruiting or will be activated soon.

### Study population

Patients aged 18–70 years with newly diagnosed CD33-positive AML with *NPM1*-mutation - without activating *FLT3* co-mutations - are eligible. Inclusion and exclusion criteria are summarized in Table 1. Patients must be considered fit for intensive chemotherapy (Eastern Cooperative Oncology Group (ECOG) performance score ≤ 2) and have adequate organ function. White blood cell count (WBC) must be < 25 × 10^9^/L with prior hydroxyurea-treatment permitted to meet this criterion.

**Table 1:** Inclusion and exclusion criteria

Inclusion criteria	1. A signed informed consent 2. Newly diagnosed, CD33-positive AML with NPM1 mutation according to WHO criteria 3. Age 18–70 years 4. Fit for intensive chemotherapy, defined by - ECOG performance status of 0–2 - Adequate hepatic function: ALAT/ASAT/Bilirubin ≤ 2.5 x ULN unless considered due to leukemic organ involvement Note: Subjects with Gilbert’s Syndrome may have a bilirubin > 2.5 × ULN per discussion between the investigator and Coordinating investigator. - Adequate renal function assessed by serum creatinine ≤ 1.5x ULN *or* creatinine clearance (by Cockcroft Gault formula) ≥ 50 mL/min 5. WBC < 25 × 10^9^/L (< 25,000/µL), prior hydroxyurea is permitted to meet this criterion 6. Ability to understand and the willingness to sign a written informed consent. 7. Male subjects must agree to refrain from unprotected sex and sperm donation from time point of signing the informed consent until 7 months after the last dose of study drug. 8. Women of childbearing potential must have a negative serum or urine pregnancy test performed within 72 h before first dose of study drug.
Exclusion criteria	1. Activating *FLT3* mutation 2. Relapsed or refractory AML 3. AML after antecedent MDS with prior cytotoxic treatment 4. Prior history of malignancy, other than MDS, unless the subject has been free of the disease for ≥ 1 year prior to start of study treatment (exceptions are basal or squamous cell carcinoma of the skin, carcinoma in situ of the cervix or of the breast, incidental histologic finding of prostate cancer (T1a or T1b using the tumor, node, metastasis clinical staging system)) 5. Previous treatment with HMA or venetoclax 6. Previous treatment for AML except hydroxyurea 7. Cumulative previous exposure to anthracyclines of > 200 mg/m^2^ doxorubicin equivalents 8. CNS involvement or extramedullary disease only 9. Known hypersensitivity to excipients of the preparation or any agent given in association with this study including venetoclax, azacitidine, cytarabine, daunorubicin, gemtuzumab- ozogamicin, or mitoxantrone 10. Known positivity for HIV and history of active or chronic infectious hepatitis unless serology demonstrates clearance of infection (i.e. PCR undetectable viral load for hepatitis) 11. Inability to swallow oral medications 12. Any malabsorption condition 13. Cardiovascular disability status of NYHA Class ≥ 2; unstable coronary artery disease (MI more than 6 months prior to study entry is permitted); serious cardiac ventricular arrhythmias requiring anti-arrhythmic therapy. *Note: Class 2 is defined as cardiac disease in which patients are comfortable at rest but* *ordinary physical activity results in fatigue*,* palpitations*,* dyspnea*,* or anginal pain.* 14. Chronic respiratory disease that requires continuous oxygen use 15. Substance abuse, medical, psychological, or social conditions that may interfere with the subject’s cooperation with the requirements of the trial or evaluation of the study results 16. Simultaneous participation in another interventional clinical trial 17. Pregnant or breastfeeding women. Breastfeeding has to be discontinued before onset of and during treatment and should be discontinued for at least 3 months after end of treatment. 18. Patients who are unwilling to follow strictly highly effective contraception requirements including hormonal contraceptives with a Pearl Index < 1% per year in combination with a barrier method from time point of signing the informed consent until 7 months after the last dose of study drug unless one of the following criteria is met: - post-menopausal (12 months natural amenorrhea or 6 months amenorrhea with serum FSH > 40 U/ml) - postoperative (6 weeks after bilateral ovarectomy with or without hysterectomy) - medically confirmed ovarian failure - vasectomy *Note: At present*,* it is not known whether the effectiveness of hormonal contraceptives is reduced by venetoclax. For this reason*,* women must use a barrier method in addition to hormonal contraceptive methods.* 19. History of clinically significant liver cirrhosis (e.g. Child-Pugh class B and C) 20. Live-virus vaccines given within 28 days prior to the initiation of study treatment *Note: corona vaccines are not live-virus vaccines and are excluded from this criterion.*

*ALAT* alanine aminotransferase, *AML* acute myeloid leukemia, *ASAT* aspartate aminotransferase, *CNS* central nervous system, *ECOG* Eastern Cooperative Oncology Group, *FLT3* FMS-like tyrosine kinase 3, *FSH* follicle-stimulating hormone, *HIV* human immunodeficiency virus, *HMA* hypomethylating agents, *MI* myocardial infarction, *MDS* myelodysplastic syndrome, NPM1 Nucleophosmin 1, *NYHA* New York Heart Association, *PCR* polymerase chain reaction, *ULN* upper limit of normal, *WBC* white blood cell count, *WHO* World Health Organization

Patients with relapsed or refractory AML, prior cytotoxic treatment, prior VEN or Hypomethylating Agents (HMA) treatment, central nervous system involvement or solitary extramedullary disease will be excluded.

The study screening period begins on the day informed consent is obtained, all screening procedures must be completed within 14 days prior to treatment initiation.

### Study treatment

In the investigational arm venetoclax will be administered orally with an initial ramp-up (100/200/400 mg) on days 1–3 of the first cycle, reaching 400 mg QD. Azacitidine will be administered subcutaneously 75 mg/m^2^ QD on days 1–7. If AZA is paused or delayed due to hematologic toxicity, VEN should be paused or delayed accordingly. Each new VEN/AZA cycle begins with the administration of AZA on day 1, provided neutrophils are ≥ 1.0 × 10^9^/L and platelets ≥ 75 × 10^9^/L, but not before day 29 of the previous cycle. VEN should be administered continuously from day 1 to day 28 unless dose interruptions are indicated. Dose modifications will be made according to the summary of product characteristics (SmPC) and are also indicated in case of co-administration with strong or moderate CYP3A inhibitors. Application of G-CSF is restricted to after first remission (at least CRh).

Within the SOC arm patients will receive one cycle of induction therapy consisting of cytarabine (200 mg/m^2^ CIV, days 1–7), daunorubicin (60 mg/m^2^ IV QD, days 3–5) and GO (3 mg/m^2^ IV QD; days 1, 4, 7). Patients not achieving CR after IT1 will be treated with age-adapted HAM, which includes cytarabine (1000–3000 mg/m^2^ IV BID, days 1–3) and mitoxantrone (10 mg/m^2^ IV QD, days 3–5). For post-remission therapy, patients will receive a maximum of three cycles of age-adapted IDAC (cytarabine 1000–1500 mg/m^2^ IV BID, days 1–3). Dose reductions are required if significant neuro-, hepato- or nephrotoxicities occur. Infusion-related reactions with GO may require dose adjustments or treatment interruptions.

### Outcomes

The primary endpoint is the modified event-free survival (mEFS). Events are defined as the following: death, primary induction failure either after a maximum of two induction cycles in the SOC arm or three induction cycles in the investigational arm, hematologic relapse after previous CR/CRi/CRh, or molecular failure defined by molecular progression (defined as ≥ 1 log_10_ increase of *NPM1* MRD level confirmed in any two samples in a patient without prior MRD negativity) or molecular relapse after previous MRD negativity (defined as confirmed ≥ 1 log_10_ increase of *NPM1* MRD level between two consecutive samples in a patient who was previously tested MRD negative).

Secondary endpoints include tolerability characterized by the cumulative occurrence of grade 3 and 4 adverse events classified as per Common Terminology Criteria for Adverse Events (CTCAE), CR/CRi/CRh/CR_MRD−_ rates, remission kinetics, MRD (assessed by both, MFC and *NPM1* RT-qPCR, MRD kinetics (molecular response and persistence), relapse-free survival and overall survival, early mortality assessed 30 and 60 days after start of induction treatment, change in health-related quality of life (HRQoL) at months 3, 6, 12, 18 and 24 and cumulative use of health care resources at 12 and 24 months (including hospital admission days, blood product usage and days on i.v. antibiotics and antifungals).

### Statistical analyses and sample size

The underlying assumption for the sample-size calculation is non-inferiority of the experimental arm VEN/AZA over intensive standard-of-care treatment. The sample size calculation was performed according to Chow et al. [14]. Assuming exponentially distributed survival times, the hazard rate λ is calculated by λ = log (2)/median survival time (MST). Schlenk et al. reported a median event-free survival time of 39.4 months in a comparable trial, corresponding to a hazard rate of 0.21 per year [15]. For this study, a hazard rate of 0.21 is assumed in both treatment arms.

The calculation assumes an accrual period of 2 years, a total trial duration of 4 years, and a loss to follow-up hazard rate of 0.2. With a significance level of 0.05, minimum power of 0.8, and a non-inferiority margin of 0.15, 136 patients (68 per arm) are required. The expected number of total events is 48.7 (24.35 per arm). To account for a 5–10% dropout rate, 10 additional patients will be enrolled, resulting in a final sample size of 146.

## Discussion

The VINCENT trial is an open-label, multi-center, phase 2 trial evaluating VEN/AZA as compared to SOC in younger, medically fit patients with treatment-naïve *NPM1*-mutated AML. While retrospective and single-arm studies have demonstrated the promising potential of VEN/AZA in various AML subgroups, this study will provide the first prospective head-to-head comparison of VEN/AZA versus SOC in patients with *NPM1-*mutated AML fit for intensive therapy. Patients with AML with *FLT3* co-mutations are excluded due to their increased risk of relapse seen in both intensively and non-intensively treated patients and the availability of effective targeted treatments, such as midostaurin, quizartinib [16–18].

The study builds on prior research suggesting that VEN/AZA achieves high rates of CRs and even MRD negativity in elderly and unfit patients and may even outperform intensive chemotherapy in patients over 65 years of age [9, 19]. The VEN/AZA regimen has been proven to have a favorable safety profile [8]. Individuals can be treated in an outpatient setting, significantly reducing costs and improving health-related quality of life.

However, the lack of prospective data in younger, fit patients has been a significant gap, which this trial seeks to address. The VINCENT trial includes a broad age range (18–70 years), making this study relevant to a broad patient population. The randomized-controlled design enables a robust evaluation of efficacy and safety of the two regimens. A large set of endpoints is assessed to ensure a comprehensive analysis of efficacy, safety and tolerability of both regimens. Close MRD monitoring (by MFC and *NPM1* RT-qPCR) allows to evaluate depth and durability of responses and prevents undertreatment. Previous studies showed a superior 2-year OS for *NPM1-*mutated AML patients achieving (bone marrow) MRD negativity in the first four VEN/HMA cycles, demonstrating a strong prognostic value of MRD diagnostics in patients treated with VEN/HMA [20].

Despite these strengths, the trial may encounter challenges. Recruiting 146 medically fit patients in this trial might be difficult given the generally low prevalence of *NPM1*-mutated, *FLT3*-wildtype AML and the emerging relevance of *NPM1* directed therapies, i.e. menin inhibitors [21, 22]. Furthermore, the open-label design, while necessary for practical reasons, may introduce potential bias, particularly for subjective endpoints like HRQoL. In the VEN/AZA arm treatment is limited to 12 cycles to standardize the comparisons with SOC. This decision is supported by recent findings indicating that discontinuing VEN/AZA therapy after achieving remission does not adversely affect patient outcomes [23, 24].

The VINCENT trial has the potential to significantly advance treatment paradigms for *NPM1*-mutated AML by providing comprehensive prospective data comparing VEN/AZA to SOC, addressing both efficacy and patient-centered outcomes.

## Trial status

Protocol version 4.0 dated 21 Jun 2023.

## Data Availability

Data (trial protocol) will be made available upon reasonable request.
